# One Patient’s Journey from Amyloidosis to Transplant

**DOI:** 10.14797/mdcvj.1151

**Published:** 2022-09-06

**Authors:** Tiffani Murphy

**Affiliations:** 1 Houston Methodist, US

**Keywords:** Light chain amyloidosis, heart and kidney transplant

## Abstract

*This story was written by a patient with immunoglobulin light chain amyloidosis who had a stem cell transplant and eventually a heart and kidney transplant. She was treated by Horacio E. Adrogue, MD, FASN, and a team of health care professionals in the Division of Nephrology, Transplantation, and Hypertension at Houston Methodist Hospital in Houston, Texas. We appreciate her willingness to share her story and hope it will inspire others*.

October 2016 is when it all started. I noticed a tiny bit of swelling in my ankles. I thought it was a little strange but that maybe I had too much salt. I elevated my legs and it was gone by morning. When I saw my primary care physician, I mentioned the swelling and also back pain and asked for labs and an x-ray, but she did not think I needed any tests since the swelling would go down overnight. I knew something wasn’t right. I had very few issues with my health and exercised regularly.

As a physician assistant, a few different diagnoses came to mind. On one end of the spectrum was systemic arthritis and at the other end was multiple myeloma, but I never said anything. It kept getting worse, and by December I had swelling up to my knees. I saw a different provider in the same office and was told it was venous insufficiency and was happening because of my age. I asked again for lab tests, but I couldn’t get them done because it was the afterhours clinic. The next week I went back and finally got the labs. At this point, I think the provider felt bad because of the delay from the week before, and after reviewing my labs, she referred me to a rheumatologist. Beginning to feel uncomfortable with this practice, I talked to a friend who referred me to an excellent rheumatologist—and this is when my life completely changed.

By the time I saw the rheumatologist in February 2017, the swelling was up to my thighs. I felt comfortable with her. She did think the swelling was excessive and added some additional labs. One of these labs came back abnormal, and I was sent to an oncologist. I will never forget how curiosity and knowledge got me to the right place. A lot of people have helped save my life, but if it wasn’t for my rheumatologist knowing the right stuff, who knows what the outcome would’ve been?

My rheumatologist referred me to a colleague, and my diagnosis was confirmed: light chain amyloidosis. This began my journey. I spent the year getting treatment to prepare me for a bone marrow transplant, which included several hospital admissions and tons of doctor appointments. I started seeing specialists in oncology, nephrology, cardiology, and transplant. The goal was to get my light chain numbers down so I could get a heart and kidney transplant. But I only had a partial response to the bone marrow transplant and needed more treatment to improve my numbers.

I spent 2018 receiving a new treatment that eventually put me in remission, but I ended up on dialysis and having a tube in my lung to drain fluid. I was emotionally drained. And to top it all off, I was denied a heart and kidney transplant due to the new development with my lung. I literally thought, “This is it, my life is over.” I had felt scared throughout this whole journey, but now I was terrified. I thought I wouldn’t see my son or my nephews growing up, and I felt everything else associated with having a rare disease. I was ready to stop fighting, but my friends insisted I keep going.

A lot happened in 2019, but the best was that my nephrologist fought for me. Earlier in the year, I had been approved for a heart transplant in California. I was about to fly back there for the kidney evaluation, but my doctors called and told me I was listed for a heart and kidney transplant in Houston. I decided not to go to California and to continue my treatment at home.

In April 2020, I got the call. Well, my son got the call because I didn’t wake up when the phone rang. I don’t remember anything about that phone call except saying, “Yes, I want the transplant.” Because of the pandemic, only my son could be with me. But we called everyone to let them know I was having the transplant. Although I was nervous, this calm feeling came over me. I knew I would be okay. My heart went out to the donor’s family because they had lost a loved one.

In preparing for the procedure, all the providers who had taken care of me from the beginning came to see me. We shared so much happiness! When the time came and I was wheeled back to the operating room, I said a prayer. When I woke up the next day, I had no pain, only relief that I would be able to get back to life.

If asked for any advice, I would say: Advocate for yourself if you know something is wrong. And don’t go through this alone—lean on your friends and family. I hope all transplant patients are as lucky as I am and have doctors who are fighters! It’s a hard fight and a long journey, but I know that if I was able to get through it, others can too.

